# Silver Nanoparticles Compromise Neurodevelopment in PC12 Cells: Critical Contributions of Silver Ion, Particle Size, Coating, and Composition

**DOI:** 10.1289/ehp.1002337

**Published:** 2010-09-14

**Authors:** Christina M. Powers, Appala R. Badireddy, Ian T. Ryde, Frederic J. Seidler, Theodore A. Slotkin

**Affiliations:** 1 Department of Pharmacology and Cancer Biology, Duke University Medical Center, Durham, North Carolina, USA; 2 Department of Civil and Environmental Engineering, Pratt School of Engineering, Center for the Environmental Implications of NanoTechnology (CEINT), Duke University, Durham, North Carolina, USA

**Keywords:** acetylcholine, developmental neurotoxicity, dopamine, in vitro, metal neurotoxicity, nanoparticles, PC12 cells, silver

## Abstract

**Background:**

Silver exposures are rising because of the increased use of silver nanoparticles (AgNPs) in consumer products. The monovalent silver ion (Ag^+^) impairs neurodevelopment in PC12 cells and zebrafish.

**Objectives and methods:**

We compared the effects of AgNPs with Ag^+^ in PC12 cells for neurodevelopmental end points including cell replication, oxidative stress, cell viability, and differentiation. First, we compared citrate-coated AgNPs (AgNP-Cs) with Ag^+^, and then we assessed the roles of particle size, coating, and composition by comparing AgNP-C with two different sizes of polyvinylpyrrolidone-coated AgNPs (AgNP-PVPs) or silica nanoparticles.

**Results:**

In undifferentiated cells, AgNP-C impaired DNA synthesis, but to a lesser extent than an equivalent nominal concentration of Ag^+^, whereas AgNP-C and Ag^+^ were equally effective against protein synthesis; there was little or no oxidative stress or loss of viability due to AgNP-C. In contrast, in differentiating cells, AgNP-C evoked robust oxidative stress and impaired differentiation into the acetylcholine phenotype. Although the effects of AgNP-PVP showed similarities to those of AgNP-C, we also found significant differences in potencies and differentiation outcomes that depended both on particle size and coating. None of the effects reflected simple physical attributes of nanoparticles, separate from composition or coating, as equivalent concentrations of silica nanoparticles had no detectable effects.

**Conclusions:**

AgNP exposure impairs neurodevelopment in PC12 cells. Further, AgNP effects are distinct from those of Ag^+^ alone and depend on size and coating, indicating that AgNP effects are not due simply to the release of Ag^+^ into the surrounding environment.

The rapid growth in the commercial use of silver nanoparticles (AgNPs) is increasing silver exposure in the general population (Wijnhoven et al. 2009). AgNPs are incorporated into products primarily as an antimicrobial, reflecting their release of monovalent silver ion (Ag^+^) (Wijnhoven et al. 2009). However, the same mechanisms that make Ag^+^ an antimicrobial also render it a potential developmental neurotoxicant. Silver crosses the placenta and concentrates in the human fetus, achieving higher concentrations than in the mother (Lyon et al. 2002). Animal studies show accumulation in the developing brain, developmental dysmorphology, and behavioral changes in exposed adults (Rungby 1990). Importantly, AgNP exposure via either inhalation or oral routes also leads to Ag accumulation in the adult rodent brain (Wijnhoven et al. 2009), altering the expression of genes involved in neuronal function (Rahman et al. 2009). We recently showed that in PC12 cells, a well-established model of neuronal development, Ag^+^ disrupts key mechanisms involved in cell replication and neurodifferentiation (Powers et al. 2010a); we then demonstrated that nervous system development is disrupted in developing zebrafish exposed to Ag^+^ (Powers et al. 2010b). Unlike primary neuronal cultures, PC12 cells provide a homogeneous population that continues to divide until differentiation is triggered by addition of nerve growth factor. Accordingly, this model allows direct study of effects on DNA synthesis associated with cell replication, an important target of neurotoxicants; the cells then differentiate into distinct acetylcholine (ACh) and dopamine (DA) phenotypes.

It is thus critical to assess the extent to which AgNPs can elicit the same or different types of neurodevelopmental outcomes as Ag^+^. In the same PC12 model, high concentrations of AgNPs disrupt the cell membrane and impair mitochondrial function (Hussain et al. 2006) while altering gene expression related to oxidative stress (Wang et al. 2009); however, these studies were not carried out in the context of neurodifferentiation (Powers et al. 2010a). In the present study, we carried out extensive experiments on replicating and differentiating PC12 cells, comparing and contrasting the effects of AgNPs with those of Ag^+^. We then evaluated the roles of particle size, coating, and composition, issues of potential importance in assessing the toxicity of different AgNP formulations (Teeguarden et al. 2007). We chose AgNPs covering a range of particle sizes and coatings, characterizing both their physical properties in suspension as well as their biological effects. We focused first on comparisons between Ag^+^ and citrate-coated AgNPs (AgNP-C). This was followed by evaluations of the effects of different coatings and sizes, using polyvinylpyrrolidine-coated AgNPs (AgNP-PVPs), and by assessments using uncoated silica nanoparticles (SiNPs) to determine whether effects could be elicited simply by particles of the same size, regardless of the main composition constituent. Nanoparticle coatings are intended to promote stability and dispensability through surface polarity that prevents agglomeration, and the two types of coatings chosen here are common to many types of nanoparticles. Commercially available nanoparticle formulations have different ranges of sizes, which can have profound influence over their biologic activities (Teeguarden et al. 2007). Our evaluations were modeled after our earlier work on the developmental neurotoxicity of Ag^+^ (Powers et al. 2010a), focusing on antimitotic effects, inhibition of protein synthesis, oxidative stress, impaired viability, and neurodifferentiation into ACh and DA phenotypes. Here, we show that the effects of AgNPs do not reflect solely their ability to release soluble Ag^+^, but instead are influenced by specific nanoparticle characteristics that dictate different biologic outcomes.

## Materials and Methods

### Nanoparticle preparation and characterization

AgNP-C was synthesized at Duke University using established methods (Lee and Meisel 1982). We purchased AgNP-PVP and uncoated SiNPs in powder form from Nanostructured & Amorphous Materials Inc. (Houston, TX). Stock suspensions of nanoparticle powders were prepared in ultrapure water, sonicated continuously at 89–95 W power, with amplitude set at 100%, for 20 min in an ice bath using a Misonix Sonicator 4000 (QSonica LLC, Newton, CT) equipped with a 1/2 in. diameter flat titanium tip.

Both types of AgNPs were prepared in stock solutions equivalent to a nominal concentration of 1 mM Ag. For AgNP-C this concentration was achieved by using 1 mM AgNO_3_ to synthesize the particles. For AgNP-PVP, we weighed out the appropriate volume of powder corresponding to 1 mM Ag, based on the percent composition from the manufacturer’s specifications. SiNP stock suspensions were made up to have a particle concentration equivalent to that used for the 10-nm AgNPs, based on AgNP and SiNP particle volumes and densities. Table 1 describes the concentration of each stock suspension in terms of the nominal Ag concentration (concentration corresponding to the Ag concentration that would be achieved if all the Ag were freely dissolved), the concentration of particles, and the mass of particles per milliliter.

We evaluated dry particle size and morphology using transmission electron microscopy at 160 kV (Tecnai G2 Twin; FEI Company, Hillsboro, OR) by adding 10 μL of the sample to a lacey carbon/copper grid (300 mesh; Electron Microscopy Sciences, Hatfield, PA) and allowing samples to air dry. Images were analyzed using Image-Pro, version 4.5 (Media Cybernetics, Inc., Bethesda, MD). Particle size in suspension was assessed using dynamic light scattering using a CGS 3 goniometer (ALV-GmbH, Langen, Germany) equipped with a helium-neon laser (633.4 nm). Suspensions were analyzed at 25°C in 5-mM diameter cells with the photomultiplier set to a scattering angle of 90°. The nominal Ag concentration in stock suspensions was measured using inductively coupled plasma-optical emission spectroscopy (Prism ICP High Dispersion; Teledyne Leeman Labs, Hudson, NH) and graphite flame atomic absorption (PerkinElmer, Waltham, MA). For PVP-coated particles, we measured the polymer concentration by baking the particles at 540°C for 18 hr in a muffle furnace; the pure silver weight was calculated from the weight of residual silver oxide formed at the end of baking. The concentration of any free PVP was assessed using a total organic carbon analyzer (TOC-5050A; Shimadzu, Columbia, MD).

### Cell cultures and assays

All materials and cell culture and assay techniques used in this study have been reported previously and were specifically used in our earlier study of Ag^+^ effects in the PC12 model (Powers et al. 2010a); therefore, we will provide only a brief procedural outline here. For studies in the undifferentiated state, the medium was changed 24 hr after seeding to include test reagents. For studies in differentiating cells, 24 hr after seeding, the medium was changed to include nerve growth factor, and each culture was examined under a microscope to verify the subsequent outgrowth of neurites. Test agents were added concurrently with the start of nerve growth factor treatment, and cultures were maintained for up to 6 days, with the indicated agents included with every medium change (48-hr intervals).

Cells were harvested and washed, and the DNA, total protein, and membrane protein fractions were isolated and analyzed as described previously (Song et al. 1998). Because neuronal cells contain only a single nucleus (Winick and Noble 1965), measuring the DNA content in each dish provides a measure of cell number. The protein/DNA ratio was calculated as an index of cell size, and the membrane/total protein ratio was used to assess the rise in membrane complexity that accompanies neurite outgrowth during neurodifferentiation. We measured DNA synthesis by assessing [^3^H]thymidine incorporation into the DNA fraction (Song et al. 1998); similarly, protein synthesis was assessed by incorporation of [^3^H]leucine into the protein fraction. Oxidative stress was evaluated through measuring the formation of lipid peroxides by reaction of the resultant malondialdehyde (MDA) with thiobarbituric acid (Qiao et al. 2005). Cell viability was measured by blinded cell counts after trypan blue staining. Differentiation into ACh and DA phenotypes was determined enzymatically by measuring choline acetyltransferase (ChAT) and tyrosine hydroxylase (TH) activities, respectively (Lau et al. 1988; Waymire et al. 1971). We also included samples containing Ag^+^ to serve as a positive test compound for comparison with the effects of the AgNPs; these represent new values, not a restatement of our published results with Ag^+^ (Powers et al. 2010a). The time points for differentiating cells were chosen based on our prior work with Ag^+^, showing progressive loss of DNA content over a span of 4–6 days in culture and changes in neurotransmitter phenotype at the 6-day point (Powers et al. 2010a). Loss of viability and oxidative stress produce eventual cell loss, so those measurements were made at 4 days.

We incorporated a number of different controls in our cell culture assays to account for differences specific to each type of nanoparticle or experimental condition, and these are described in the figure legends. In our previous study (Powers et al. 2010a) we found no effect of nitrate ion on any of these parameters and thus did not include this additional control in the present study. Similarly, we did not include citrate controls because the culture medium already contains citrate in substantial concentrations from the added fetal bovine and horse serum.

### Data analysis

All studies were performed on 8–16 separate cultures for each measure and treatment, using 2–4 separate batches of cells. Results are presented as mean ± SE. Treatment effects were established by analysis of variance (ANOVA), followed by Fisher’s protected least significant difference test for post hoc comparisons of individual treatments; data were log-transformed whenever the variance was heterogeneous. In the initial test, we evaluated two ANOVA factors (treatment and cell batch) and found that the results did not vary among the different batches of cells, so results across the different batches were normalized and combined for presentation. Significance was assumed at *p* < 0.05 (two-tailed).

## Results

### Nanoparticle characteristics

**Figure 1:**
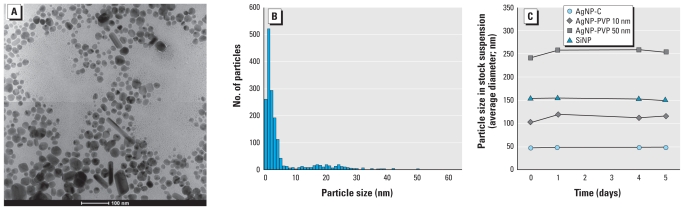
Nanoparticle characteristics. (*A*) Transmission electron microscopy of AgNP-C, showing both unaggregated and aggregated nanoparticles. (*B*) Distribution of AgNP-C dry particle sizes. (*C*) Dynamic light scattering measurements of particle diameter in stock suspensions (mean of 20 measurements), showing slight aggregation for all particles.

The vast majority of nanoparticles were spherical. AgNP-C was polydisperse (Figure 1A), with an average dry particle size of 6 nm; 85% were < 10 nm, and the remaining 15% were < 63 nm (Figure 1B). In suspension, particles swelled or aggregated, producing a higher hydrodynamic radius compared with dry particles. The size remained stable over time (Figure 1C), indicating either that aggregated particles fell out of suspension or that they represented only a small proportion. We examined the particle concentration under culture conditions over the 48-hr time period between medium changes, using spectrophotometry at 540 and 570 nm to assess absorption by the particles suspended in the cell culture medium, focusing on the highest concentration (100 μM nominal Ag), which would be most likely to aggregate. The suspended nanoparticle concentration remained unchanged over 48 hr: 0.030 optical density units above culture medium alone at 24 hr, and 0.034 at 48 hr (triplicate samples). Thus, particles in suspension tended to aggregate somewhat over time but maintained their average size and concentration, indicating that aggregation was not a significant problem.

We carried out similar evaluations of AgNP-PVP. Dry particles designated to have a 10-nm diameter actually averaged 21 nm, with 88% at < 25 nm and the remainder at < 200 nm. The designated 50-nm AgNP-PVP actually averaged 75 nm, with 57% at < 81 nm and the remainder at < 200 nm. Our analysis of dry SiNP showed good agreement with the manufacturer’s stated description of particle size and shape. Similar to AgNP-C, both PVP-coated particles and SiNP showed a small degree of aggregation once in suspension, but the effects were of insufficient magnitude to cause major changes in the suspended nanoparticle concentration (Figure 1C). We also used a total organic carbon analyzer to measure the PVP concentration in the AgNP-PVPs. We found PVP concentrations of 15% and 13% of total AgNP weight for 10- and 50-nm particles, respectively, markedly higher than the stated concentrations of 0.2–0.3%. In preparing our stock concentrations, we used 10% PVP (i.e., between our values and the manufacturer’s). The measured values for Ag in the dry Ag-PVPs were within 15% of those expected.

Throughout the results, we present the nanoparticle concentration in two different metrics: the nominal Ag concentration (defined as the equivalent of all the Ag being in free solution) and the number of particles per unit volume. The equivalent amount for each nanoparticle (mass per unit volume) appears in Table 1.

### AgNP-C in undifferentiated cells

**Figure 2:**
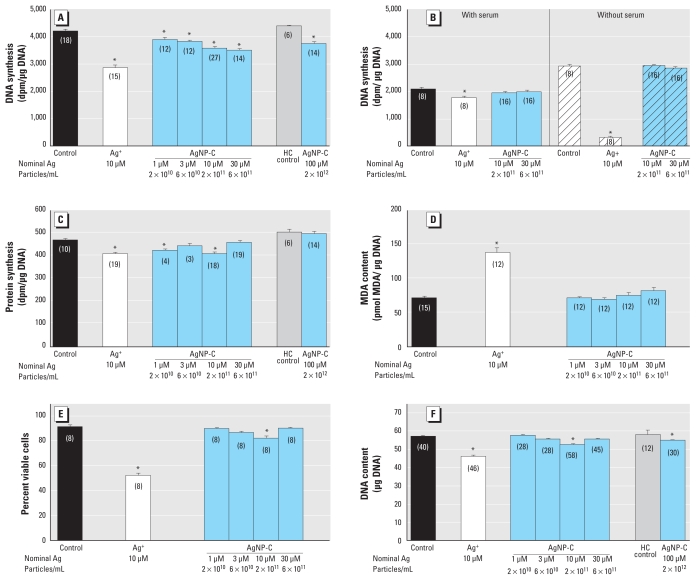
Effects of AgNP-C on undifferentiated PC12 cells shown by (*A*) DNA synthesis (24-hr exposure), (*B*) DNA synthesis in the presence or absence of serum (1 hr exposure), (*C*) protein synthesis (24-hr exposure), (*D*) oxidative stress (24-hr exposure), (*E*) trypan blue exclusion (24-hr exposure), and (*F*) DNA content (24-hr exposure). Data represent mean ± SE of the number of determinations shown in parentheses. ANOVA for each panel indicated a main treatment effect [*p* < 0.0001; (*A*) F_7,110_ = 9.2, (*B*) F_7,88_ = 123, (*C*) F_7,87_ = 8.7, (*D*) F_5,69_ = 32, (*E*) F_5,42_ = 44, (*F*) F_7,249_ = 6.1]. To achieve 100 μM AgNP-C, the culture medium was diluted 10% with AgNP-C stock solution; isotonic NaCl and NaHCO_3_ were then added to achieve isotonicity and to match the NaHCO_3_ concentration normally in the medium; accordingly, these samples have separate controls with the same additions. HC control, high concentration control. *Significantly different from the corresponding control (*p* < 0.05 or better) by Fisher’s protected least significant difference test.

We first compared the antimitotic effects of AgNP-C with those of Ag^+^. With a 24-hr exposure, we found a concentration-dependent decrease in DNA synthesis starting at AgNP-C corresponding to a nominal Ag concentration of 1 μM, but in all cases the effects were smaller than those seen with 10 μM Ag^+^ (Figure 2A). To determine if binding of AgNPs to serum proteins was responsible for the smaller effect compared with Ag^+^, we measured DNA synthesis in cells exposed to AgNP-C or Ag^+^ with and without serum for 1 hr, a span in which cells maintain their viability in the absence of serum (Figure 2B). Removing serum from the medium greatly enhanced the effect of Ag^+^, reflecting a high degree of binding to serum proteins. However, there was no corresponding enhancement for AgNP-C.

To determine if the reduction in DNA synthesis evoked by AgNP-C reflected a specific action on mitotic activity, we examined corresponding effects of a 24-hr exposure on protein synthesis (Figure 2C). For Ag^+^, the reduction in protein synthesis was much smaller than that seen for DNA synthesis. In contrast, for AgNP-C, protein synthesis was inhibited to about the same extent as had been observed for its effects on DNA synthesis up to a nominal Ag concentration of 10 μM. However, unlike the situation for DNA synthesis, the effect on protein synthesis was lost at higher concentrations.

In undifferentiated cells, Ag^+^ produced robust oxidative stress after a 24-hr exposure, whereas AgNP-C was ineffective (Figure 2D). Likewise, Ag^+^ was much more cytotoxic, evoking a large reduction in cell viability compared with the much smaller effect of AgNP-C (Figure 2E); similar to the effect on protein synthesis, AgNP-C above a nominal Ag concentration of 10 μM became less effective. Finally, measures of cell number after a 24-hr exposure (DNA content) confirmed that Ag^+^ evoked a much greater cell loss than did AgNP-C (Figure 2F) and, again, we saw a nonmonotonic effect of the nanoparticles.

### AgNP-C in differentiating cells

**Figure 3:**
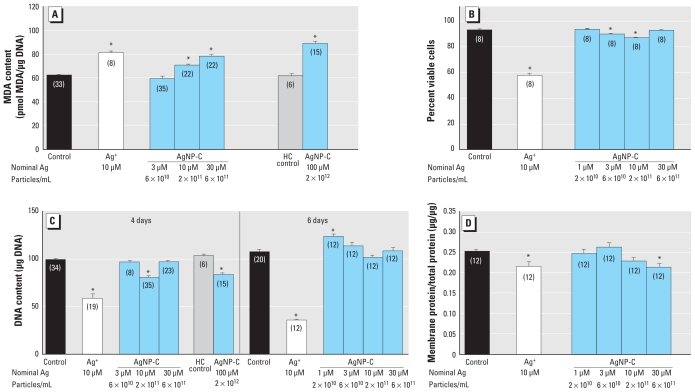
Effects of AgNP-C on differentiating PC12 cells shown by (*A*) oxidative stress (4-day exposure), (*B*) trypan blue exclusion (4-day exposure), (*C*) DNA content (4 and 6-day exposures), and (*D*) membrane/total protein ratio (6-day exposure). Data represent mean ± SE of the percent change from control, based on the number of determinations shown in parentheses. ANOVA for each panel indicated a main treatment effect [*p* < 0.01 or better; (*A*) F_6,134_ = 47, (*B*) F_5,42_ = 65, (*C*) F_6,133_ = 37 at 4 days and F_5,74_ = 107 at 6 days, (*D*) F_5,66_ = 3.6]. HC control, high concentration control. *Significantly different from the corresponding control (*p* < 0.05 or better) by Fisher’s protected least significant difference test.

Unlike the situation in undifferentiated cells, AgNP-C exceeding a nominal Ag concentration of 3 μM produced significant oxidative stress after 4 days of exposure, in the same range as Ag^+^ (Figure 3A). Increased oxidative stress was not secondary to general cytotoxicity, as the AgNP-C effect on viability remained much smaller than that of Ag^+^ (Figure 3B); furthermore, although the dose–effect relationship was monotonic for oxidative stress, it was nonmonotonic for loss of viability. At the same 4-day exposure, cell number decreased much more at 10 μM Ag^+^ than at comparable or higher concentrations of AgNP-C (Figure 3C); for AgNP-C, we observed small but significant decrements at nominal Ag concentrations of 10 and 100 μM, albeit not at 30 μM. Furthermore, by 6 days of exposure, cell number recovered so that there was no detectable loss at any concentration; in fact, there was an increase in cell number at the lowest AgNP-C concentration (Figure 3C). In contrast, exposure to Ag^+^ simply produced a progressive cell loss beyond that seen at the 4-day point.

We also assessed indices of cell growth and neurodifferentiation. None of the AgNP-C concentrations produced significant changes in the total protein/DNA ratio (data not shown), an index of cell size. Nevertheless, AgNP-C had a progressive effect on the membrane/total protein ratio, an index of neurite outgrowth, achieving statistical significance at a nominal Ag concentration of 30 μM (Figure 3D); 10 μM Ag^+^ impaired neurite formation to about the same extent. A 6-day exposure to AgNP-C at a nominal Ag concentration of 30 μM clearly decreased emergence of the ACh phenotype [ChAT activity (mean ± SE): control, 109 ± 6 pmol/hr/μg DNA; AgNP-C, 69 ± 1 pmol/hr/μg DNA; *p* < 0.0001; *n* = 8) without significantly affecting the DA phenotype (TH activity: control, 118 ± 6 pmol/hr/μg DNA; AgNP-C, 110 ± 7 pmol/hr/μg DNA; not significant, *n* = 8). Accordingly, the TH/ChAT ratio rose (control, 1.09 ± 0.04; AgNP-C, 1.66 ± 0.10; *p* < 0.002), indicating a phenotype shift.

Ascorbate prevents oxidative stress and cell loss caused by exposure to Ag^+^ (Powers et al. 2010a). Accordingly, we performed complementary experiments with AgNP-C at a nominal Ag concentration of 10 μM. We found the same increase in MDA in the presence of ascorbate (mean ± SE:10 μM, 17 ± 2% increase; *p* < 0.0001; *n* = 20) and loss of DNA (20 ± 2% decrease; *p* < 0.0001; *n* = 20) as without ascorbate (Figure 3A,C).

### Effects of nanoparticle coating, size, and composition

**Figure 4:**
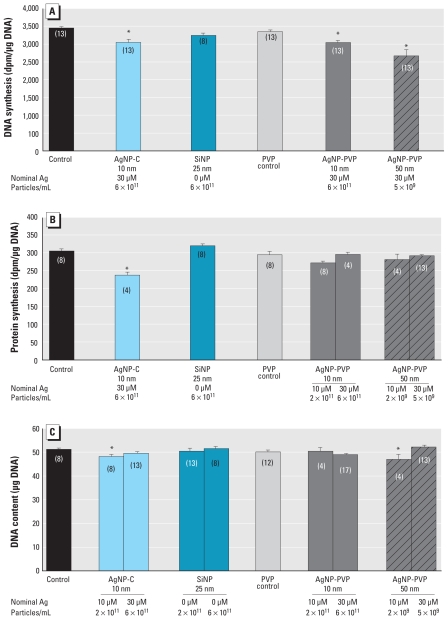
Effects of particle size, coating, and composition in undifferentiated PC12 cells after a 24-hr exposure shown by (*A*) DNA synthesis, (*B*) protein synthesis, and (*C*) DNA content. Data represent mean ± SE of the percent change from control, based on the number of determinations shown in parentheses. ANOVA for each panel indicated a main treatment effect [*p* < 0.003 or better; (*A*) F_5,67_ = 7, (*B*) F_7,50_ = 9.2, (*C*) F_9,90_ = 5.4]. The PVP control group contained the same volume of a 10% PVP stock solution as that of the AgNP-PVP. *Significantly different from the corresponding control (*p* < 0.05 or better) by Fisher’s protected least significant difference test.

In undifferentiated cells, a 24-hr exposure to AgNP-PVP with manufacturer-designated diameters of either 10 or 50 nm at a nominal Ag concentration of 30 μM evoked significant decreases in DNA synthesis (Figure 4A). Notably, the decrement for the 50-nm AgNP-PVP exceeded that caused by the smaller AgNP-PVP or by AgNP-C. SiNP had a smaller (nonsignificant) effect than any of the AgNPs. Similar measures of protein synthesis showed no discernible effect of either 10 or 50 nm AgNP-PVP at nominal Ag concentrations of 10 or 30 μM, whereas 10 μM AgNP-C clearly inhibited synthesis (Figure 4B). SiNPs had no effect. With the same 24-hr exposure to a nominal 10 μM Ag concentration, AgNPs reduced cell number, with the greatest effect from AgNP-PVP 50 nm, followed by AgNP-C, and no effect for AgNP-PVP 10 nm (Figure 4C); however, at a 30 μM nominal Ag, the effect of 50 nm AgNP-PVP or AgNP-C was reduced or lost. Again, SiNPs had no effect.

**Figure 5:**
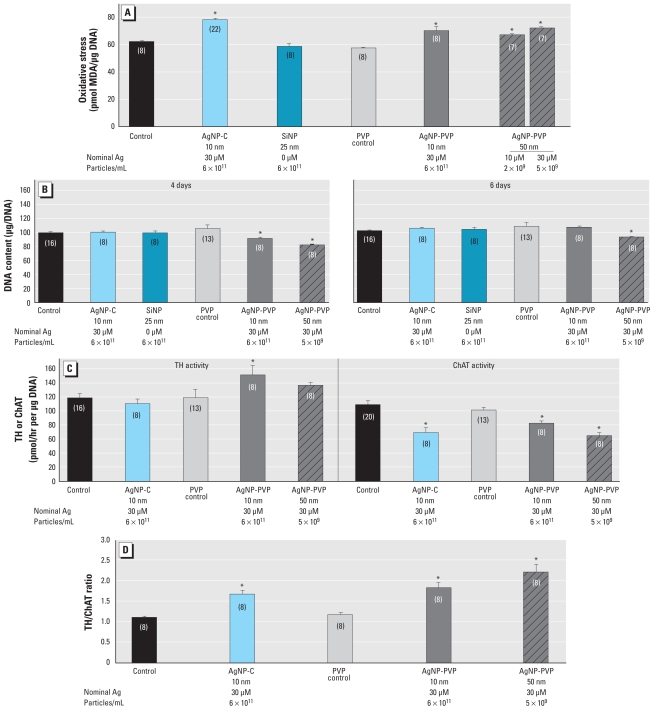
Effects of particle size, coating, and composition in differentiating PC12 cells shown by (*A*) oxidative stress (4-day exposure), (*B*) DNA content (4- and 6-day exposure), (*C*) TH and ChAT (6-day exposure), and (*D*) TH/ChAT ratio (6-day exposure). Data represent mean ± SE of the percent change from control based on the number of determinations shown in parentheses. ANOVA for each panel indicated a main treatment effect [*p* < 0.02 or better; (*A*) F_6,61_ = 15, (*B*) F_5,55_ = 15 at 4 days and F_5,55_ = 2.8 at 6 days, (*C*) F_4,48_ = 3.8 for TH and F_4,52_ = 14 for ChAT, (*D*) F_4,35_ = 15]. *Significantly different from the corresponding control (*p* < 0.05 or better) by Fisher’s protected least significant difference test.

We next compared the effects of particle size, coating, and composition in differentiating cells. With a 4-day exposure, we found oxidative stress for all three types of AgNPs, whereas SiNP was ineffective (Figure 5A). Either size of AgNP-PVP decreased cell number at 4 days (Figure 5B); by 6 days, the effect regressed to normal for the smaller AgNP-PVP particle but not for the larger particle. AgNP-C at the same nominal concentration had no measurable effect on cell number at either time point (Figure 5B, replicating the results seen in Figure 3C). Both sizes of AgNP-PVP increased the index of cell size at 6 days: 9 ± 3% (mean ± SE) increase in the total protein/DNA ratio for 10 nm AgNP-PVP (*p* < 0.01; *n* = 20), 7 ± 2% increase for 50 nm AgNP-PVP (*p* < 0.04; *n* = 20; control ratio 27.8 ± 0.4 μg/μg) but had no significant effect on the membrane/total protein ratio (data not shown). Samples with corresponding concentrations of AgNP-C run concurrently with the AgNP-PVP showed no significant difference in total protein/DNA. Finally, 10 nm AgNP-PVP enhanced differentiation into the DA phenotype, as indicated by a significant increase in TH activity relative to control values, but neither 50 nm AgNP-PVP nor AgNP-C had a comparable effect (Figure 5C). In contrast, all three types of AgNPs suppressed the ACh phenotype, as shown by deficits in ChAT activity, but AgNP-C and 50 nm AgNP-PVP were more effective than the smaller-diameter AgNP-PVP. Although the underlying components differed, all of the AgNPs increased the ratio of TH/ChAT (Figure 5D), reflecting diversion of cells toward the DA phenotype and away from the ACh phenotype; the net effect was greatest for the larger-diameter AgNP-PVP.

## Discussion

Our results provide some of the first evidence that AgNPs can act as developmental neurotoxicants in a model of neuronal cell replication and differentiation. They further point to compound effects that depend not only on the release of Ag^+^ but also on particle size, coating, and composition. Indeed, if AgNPs acted solely by releasing soluble Ag^+^, then all of their effects would resemble those of lower concentrations of the soluble ion, because a large proportion of the Ag^+^ is not dissolved. In that case, we would expect to see a decline in AgNP effect with increasing particle size because the smaller surface-to-volume ratio of larger particles would render less of the Ag^+^ available to dissolve. Some of our findings followed this predicted pattern, but others clearly did not. The effects of AgNP-C on undifferentiated cells were in the same direction but decidedly smaller than those from the same concentration of freely dissolved Ag^+^ for comparisons of DNA synthesis, oxidative stress, viability, and cell loss. However, this was not true for the role of plasma protein binding on DNA synthesis, nor for the effects on protein synthesis; for the latter, AgNP-C was as effective as Ag^+^ and showed a nonmonotonic effect that was not seen in our earlier work with Ag^+^ (Powers et al. 2010a). The same pattern of some similarities, coupled with important dichotomies, was apparent in differentiating cells. AgNP-C produced oxidative stress, decreased viability, cell loss, and impaired neurite formation, in each case requiring a higher concentration to produce effects equivalent to those seen from the freely dissolved Ag^+^. Likewise, in our earlier work with Ag^+^, we found a biphasic effect on DNA content between 4 and 6 days of exposure just as seen here for AgNP-C, but involving a lower Ag^+^ concentration. Nevertheless, AgNP-C failed to evoke the cell enlargement (increased total protein/DNA) seen with Ag^+^; furthermore, ascorbate did not protect cells from the oxidative stress and cell loss caused by AgNP-C, whereas the same treatment protects against Ag^+^ (Powers et al. 2010a). Finally, the experiments using the two sizes of AgNP-PVP showed a relationship opposite what would be expected just from release of Ag^+^ from the particle surface: At the same nominal Ag concentration, the larger nanoparticle had greater effects on DNA synthesis and content and caused a higher degree of disruption in oxidative stress and neurotransmitter phenotype.

Clearly, then, the neurotoxic actions of AgNPs involve significant contributions from nanoparticle formulation, albeit not from only the physical dimensions, because SiNPs were generally ineffective in producing the effects seen with the AgNPs. Nanoparticles can produce their unique effects either through altering access to the interior of the cell (pharmacokinetic effects) or through eliciting responses that differ from those of the freely dissolved materials (pharmacodynamic effect). Our results for effects on DNA synthesis with and without serum effectively eliminate the possibility that a reduced effect of AgNPs results from binding to serum proteins. In fact, we found the opposite, namely, that removal of serum greatly enhanced the effect of Ag^+^ but not that of AgNP-C. Similarly, we can rule out the possibility that nanoparticle aggregation limits the concentration of Ag^+^ available for biologic effects for two reasons. First, we saw little evidence for significant changes in the net nanoparticle size or concentration over time. Second, aggregation would produce a parallel change in all the measured effects, whereas we saw a monotonic concentration–response curve for some variables but nonmonotonic effects for others.

Instead, our results provide conclusive evidence for unique biologic effects of AgNPs, distinct from the actions of freely dissolved Ag^+^ and unrelated to simple pharmacokinetic attributes. Four key findings support this interpretation:

- The lack of AgNP-C selectivity toward DNA versus protein synthesis in undifferentiated cells (whereas Ag^+^ is highly selective toward the former macromolecule)
- The inability of ascorbate to protect cells from oxidative stress and cell loss caused by AgNP-C (whereas the same antioxidant is protective against Ag^+^) (Powers et al. 2010a), which implies that cell loss from AgNP-C reflects a different underlying mechanism and that, for the nanomaterial, oxidative stress is a result of cytotoxicity, not a cause of it
- The greater inhibition of protein synthesis at lower AgNP-C concentrations and a loss of effect at higher concentrations, totally distinct from the monotonic dose–effect relationship for Ag^+^ (Powers et al. 2010a), which indicates that low AgNP-C concentrations disrupt protein synthesis through a mechanism unrelated to freely dissolved Ag^+^
- The restricted effect of AgNP-C to suppress the ACh phenotype (Ag^+^ affects both ACh and DA phenotypes) (Powers et al. 2010a).

A comparison of AgNP-C with the two sizes of AgNP-PVP readily illustrates the roles of nanoparticle coating and size. Particle coatings clearly affected biological outcomes: One or both PVP-coated particles had greater effects than AgNP-C toward cell loss, cell size, and promotion of TH activity, yet the AgNP-PVPs had no effect on protein synthesis. If the coating simply altered the dissolution of Ag^+^, then all the comparative effects would have been similar. At the same time, the larger-diameter AgNP-PVP had greater effects than the smaller nanoparticles on most of the outcomes. Studies with gold nanoparticles show that particles ≥ 50 nm are actively taken up into cells, whereas smaller particles are not (Johnston et al. 2010), thus providing a possible explanation for the generally greater effects seen here for the larger AgNP-PVP. However, this was not the case for the effects on differentiation into neurotransmitter phenotypes, where the 10-nm AgNP-PVP had promotional effects on DA greater than those obtained with the 50-nm nanoparticle. Thus, AgNPs not only elicit effects distinct from those of Ag^+^, but also display important differences that are dictated by particle size and coating and specific to each biological process. This finding strongly indicates that AgNPs act biologically as nanoparticles and not just as a source of Ag^+^.

Although we present strong evidence that AgNPs work through a combination of Ag^+^ release and mechanisms that reflect actions of the AgNPs themselves, more work is clearly needed to understand the mechanisms underlying nanoparticle effects and the interactions of nanoparticles with extracellular and intracellular components. With regard to the former, our data show that immediate, antimitotic effects of AgNPs are not sensitive to the presence of serum proteins, but it is certainly likely that interactions could occur with the more prolonged exposures that would occur *in vivo* or as particles interact with proteins on the cell surface. Indeed, addition of serum mitigates the loss of viability during a 24-hr exposure to AgNPs in mouse keratinocytes (Murdock et al. 2008). Secondly, the diminished effects of AgNP-C versus Ag^+^ toward oxidative stress in undifferentiated cells and toward viability in either differentiation state, as well as nonmonotonic effects for these and other variables, may reflect protective actions of the citrate coating. Soluble citrate could supplement cellular metabolic and biosynthesis demands (Bauer et al. 2005), thereby ameliorating effects of AgNP exposure. This could also explain why the 50-nm AgNP-PVP, despite its larger size, had generally greater effects than AgNP-C at the same nominal Ag concentration.

Given the rapid growth in AgNP use (Wijnhoven et al. 2009), detailed studies of the biological effects on neurodevelopment are critically important. *In vitro* models, such as that used here, can guide future *in vivo* studies to focus on critical stages of neuronal vulnerability, such as neurotransmitter targets and underlying cellular mechanisms. Our findings point to the likelihood that AgNPs are developmental neurotoxicants that will display a wide window of vulnerable stages, ranging from events in early development (mitosis, cell survival) through later stages of neurodifferentiation. The nonmonotonic dose–response relationships seen with the nanoparticles, along with the dependence on coating and size, point to multiple mechanisms of action rather than a single mechanism. Accordingly, the effects of *in vivo* exposures may differ substantially at different developmental stages and at different ends of the dose–response continuum. One uniform finding, however, was that the AgNPs, like Ag^+^, divert the end phenotype away from ACh and toward DA, albeit by different contributory mechanisms for each individual agent. If this occurs *in vivo*, we would expect to find substantial miswiring of ACh and DA circuits. Accordingly, we would then predict that the specific neurobehavioral outcomes of AgNP exposure are likely to include adverse effects on cognitive, reward, and motor performance. We are currently examining outcomes of AgNP exposure in developing zebrafish to determine if, as predicted by these *in vitro* studies, AgNPs are developmental neurotoxicants *in vivo*.

## Figures and Tables

**Table 1:** Nanoparticle stock suspension concentrations.

Measure	AgNP-C 10 nm	AgNP-PVP 10 nm	AgNP-PVP 50 nm	SiNP 25 nm
Nominal Ag (mM)	1	1	1	0
Particles per milliliter	2 × 10^13^	2 × 10^13^	3 × 10^11^	2 × 10^13^
Micrograms per milliliter	108	108	108	374
